# *NetPlotBrain*: A Python package for visualizing networks and brains

**DOI:** 10.1162/netn_a_00313

**Published:** 2023-06-30

**Authors:** Silvia Fanton, William Hedley Thompson

**Affiliations:** Department of Clinical Neuroscience, Karolinska Institutet, Stockholm, Sweden; Department of Applied Information Technology, University of Gothenburg, Gothenburg, Sweden

**Keywords:** Network visualizations, Python, Network neuroscience, Research software

## Abstract

Visualizations of networks are complex since they are multidimensional and generally convey large amounts of information. The layout of the visualization can communicate either network properties or spatial properties of the network. Generating such figures to effectively convey information and be accurate can be difficult and time-consuming, and it can require expert knowledge. Here, we introduce *NetPlotBrain* (short for *network plots onto brains*), a Python package for Python 3.9+. The package offers several advantages. First, *NetPlotBrain* provides a high-level interface to easily highlight and customize results of interest. Second, it presents a solution to promote accurate plots through its integration with *TemplateFlow*. Third, it integrates with other Python software, allowing for easy integration to include networks from *NetworkX* or implementations of network-based statistics. In sum, *NetPlotBrain* is a versatile but easy to use package designed to produce high-quality network figures while integrating with open research software for neuroimaging and network theory.

## Author Summary

*NetPlotBrain* is a Python package to easily create network visualizations on a brain and view brain anatomy. *NetPlotBrain* is integrated with *TemplateFlow* and popular Python packages, the former facilitating the selection of the appropriate template or atlas from the available options and the latter providing the user with easy customization and fine-tuning.

## INTRODUCTION

Visualizations in science display various types of information, ranging from theoretical models to empirical data (Sargent, 1996). They are critical for effective communication of quantitative information (Cheng et al., 2022; Tufte, 1985; van Wijk, 2006) and for facilitating the learning of scientific information (Nolan & Perrett, 2016; Rom, 2015; Vavra et al., 2011). Visualizations also have a crucial role in exploratory science, which is a central method to learn from data (Behrens, 1997; Fox & Hendler, 2011; Tukey, 1977). One trend within some programmatic visualization tools has aimed to provide users with high-level interfaces. This trend entails that users specify and customize their plot by only pointing to the data, and the visualization tool will then handle the rest. Alternatively, for low-level interfaces, users specify individual aspects of the figure or input each variable independently. Frequently, high-level interfaces are implemented by inputting a data frame (i.e., a table of data with column names). Users then specify different column names for aspects of the figure they would like the visualization tool to manipulate. Examples of high-level user visualization interfaces can be seen in tools such as *GGPlot2* for *R* (Wickham, 2016) and *Seaborn* for Python (Waskom et al., 2020).

Visualizing networks is challenging because the visualization represents complex multidimensional topologies embedded in the network within the two-dimensional media of a static figure. For instance, a commonly used format in network neuroscience involves circular layouts, where all the nodes are placed in a circle. Alternatively, other representations include spring, spectral, or random layouts depending on the choice of node placement algorithm that leverages the network features such as structure. Algorithms for displaying networks in informative ways have become a field itself (see McGuffin, 2012). When the location of the nodes is important, as is often the case with brain networks, nodes are often placed in the coordinate system corresponding to their location (e.g., the brain). There are many tools available today to achieve these different types of visualizations. For visualizing networks in the brain, there is *BrainNet Viewer* (Xia et al., 2013) and *nilearn* (Abraham et al., 2014). For general network layouts, there is *Cytoscape* (Smoot et al., 2011), *Gephi* (Bastian et al., 2009), *NetworkX* (Hagberg et al., 2008), *Circos* (Krzywinski et al., 2009), and *igraph* (Csárdi & Nepusz, 2006). Additionally, for other types of visualizations of multimodal brain data, there are *Brainrender* (Claudi et al., 2021), *Visbrain* (Combrisson et al., 2019), and *PySurfer* (https://pysurfer.github.io/).

All tools have trade-offs regarding their detail, ease of use, and suitability for certain questions and use cases. However, we identified three features that we would like to see within a single tool for brain network visualizations. The tool should do the following:

- be easy to specify and customize
- have easy integration with software relating to neuroimaging, but also with more general network software
- promote the user to plot accurate templates concerning the coordinate system of the nodes

While some currently available software attempt to solve these different points to varying degrees, we do not believe any yet sufficiently solves all three. To solve the problem, we developed *NetPlotBrain* (*network plots onto brains*), a Python 3.9+ package. It is built on scientific Python libraries, including *Matplotlib*, *pandas*, and *NumPy*, while also being compatible with more specific packages such as *NetworkX* and other Python implementations of network-based statistics. The high-level interface is built up as a single function with an array to tailor the visualization to one's needs. Finally, it leverages *TemplateFlow* (Ciric et al., 2022) to provide access to a large and expanding portfolio of brain templates and atlases, with complete provenance and versioning records that ensure accuracy and reproducibility.

This article will proceed by introducing how to use different aspects of *NetPlotBrain* (Thompson et al., 2023; https://github.com/wiheto/netplotbrain). First, we discuss the basic workflow and input of data. Second, we discuss how figures can be customized, including the high-level interface. Third, we show examples of how *NetPlotBrain* integrates with other software. Finally, the Methods section discusses installation and dependencies.

## RESULTS

Replicating programming patterns of more general-purpose visualization utilities such as *Matplotlib*, the core function of *NetPlotBrain*’s user interface is netplotbrain.plot().

### A User Interface Optimized for the Visualization of Human and Nonhuman Brain Networks

Users specify three different data inputs to display brain networks. These three inputs are (a) the template, which specifies an anatomical image and a 3D coordinate system; (b) the nodes, which specify how the spatial frame established by the template is partitioned to define nodes; and (c) the edges, which give rise to the specific connectivity. Each of these components has a keyword argument that can be passed:

fig, ax = netplotbrain.plot(template=…, nodes=…, edges=…).

All three arguments are optional (i.e., it is possible to plot a template by itself or just plot the nodes by themselves). If edges are specified, the “nodes” argument must be specified.

The two outputs of the function are objects of *Matplotlib* classes. The first of these objects (fig) is of the Figure class for *Matplotlib*. The second is a list of *Matplotlib* 3D axes (Axes3D) for each of the subplots. These outputs allow the users to further specify low-level properties of the plot at will with *Matplotlib*. However, the users do not need to interact with these outputs, if they do not wish, as they can save the figure directly (as a .png or .svg file) in the *NetPlotBrain* function using the “savename” keyword argument.

**Figure 1.**
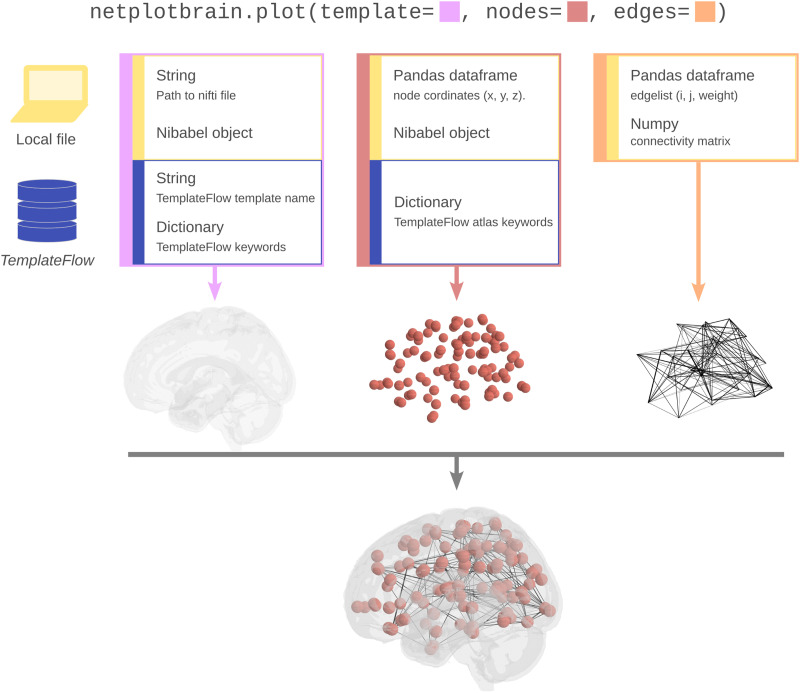
Summary of the different input formats and file locations for the different *NetPlotBrain* components. Information regarding templates, nodes, and edges can be provided locally or from *TemplateFlow*. Each of the three components can be plotted and customized independently.

The respective input to each of these three arguments can be specified in multiple ways to maximize flexibility. Spatial inputs (i.e., the template and the nodes) can originate locally or be procured by *TemplateFlow* (Ciric et al., 2022). *TemplateFlow* is an online repository of MRI atlases and templates that unambiguously names standard neuroimaging spaces and related resources (e.g., templates and atlases). We will discuss each data component and outline how that data can be submitted (see Figure 1).

#### The spatial reference of the visualization—the template.

The template shows the brain as a background image for the nodes and edges to be plotted onto. Its function is to help the user see where the different nodes are located in the brain. For different purposes and tastes, some may want a more detailed outline, while others may want just the contours of the brain.

Primarily, the template can be specified as a string containing template names of MRI images available on templateflow.org:

netplotbrain.plot(template='MNI152NLin2009cAsym').

Conveniently, the *TemplateFlow* client implements *lazy-loading*, and the template file will be downloaded only once upon the first use onto the user’s local hard drive. *NetPlotBrain* will attempt to take the most suitable anatomical image for the requested template. By default, *NetPlotBrain* seeks a segmented T1w image of the brain. If that file is not present for a template space, then *NetPlotBrain* takes the binary mask of the segmented brain. Further flexibility and options in template selection (e.g., when the selected template features multiple cohorts) are comprehensively described in the documentation.

Alternatively, the template can be any skull-stripped or segmented three-dimensional NIfTI (Neuroimaging Informatics Technology Initiative; Cox et al., 2004) image file stored locally, specified either as a string containing the path in a file system,

netplotbrain.plot(template='./path/to/img.nii.gz'),

or as a *nibabel* object,

import nibabel as nib

…

img=nib.load('./path/to/img.nii.gz')

netplotbrain.plot(template=img).

This alternative template specification allows, for instance, the visualization of networks corresponding to a specific, individual brain or standard spaces defined by templates unavailable within *TemplateFlow* (e.g., customized, study-wise templates or templates with reuse restrictions that cannot be shared openly).

#### Defining the nodes.

Nodes represent the different brain regions. Nodes can be specified as a *pandas DataFrame* of coordinates, a NIfTI image indicated as a string, a *nibabel* object stored locally, or a dictionary with key/value pairs of any *TemplateFlow* atlas available on templateflow.org.

When nodes are specified as a *pandas DataFrame*, node information should be contained in columns 'x', 'y', 'z' or identified using the “node_columnnames” argument:

import pandas as pd

…

node_df = pd.DataFrame(data={'x':               […],

               'y':               […],

               'z':               […]})

netplotbrain.plot(nodes=nodes_df).

The missing input in the 'x', 'y', and 'z' columns consists of lists of coordinates in the respective template space. Additional columns in the *pandas DataFrame* can be added to contain additional data to be used in the figure (see the *High-level interface* subsection below). The default column names for coordinates can be changed with the “node_columnnames” keyword argument.

Nodes can also be defined by a 3D NIfTI map containing a discrete (i.e., piecewise smooth) partition of the reference brain defined by the template. The partition is discrete because a unique integer label identifies each node. Here, the alternatives are similar to the local template input, where the input is a string that designates a path to a NIfTI file,

netplotbrain.plot(nodes='./path/to/img.nii.gz'),

or a *nibabel* object,

import nibabel as nib

…

img         =        nib.load('./path/to/img.nii.gz')

netplotbrain.plot(nodes=img).

The “nodes” argument can also point to a *TemplateFlow* atlas. An atlas can be selected by specifying a dictionary of keyword/value options to choose the template. For example, the following arguments in netplotbrain.plot will download the 400 parcel parcellation from the Schaefer2018 atlas (Schaefer et al., 2018) in the MNI152NLin2009cAsym space from *TemplateFlow*:

nodes_tf = {'atlas':    'Schaefer2018',

      'desc':    '400Parcels7Networks',

      'resolution': 1}.

netplotbrain.plot(template='MNI152NLin2009cAsym', nodes=nodes_tf).

#### Connecting the nodes—the edges.

Edges specify connections between nodes. Edges can be expressed as either a *NumPy* array (adjacency matrix) or a *pandas DataFrame* (edge list).

When a *NumPy* array is used, the array should be *N* × *N* in shape, where *N* denotes the number of nodes. The below code will plot random edges for 100 predefined nodes:

import numpy as np

…

netplotbrain.plot(nodes=nodes,

          edges=edges_array),

where the edges_array variable contains a 100 × 100 *NumPy* array.

When a *pandas DataFrame* is used, edge information should be specified in the default columns 'i' and 'j', whereas 'weight' can be an optional column for edge width.

import pandas as pd

…

edges_df = pd.DataFrame(data={'i':              […],

               'j':              […],

               'weight':            […]}).

netplotbrain.plot(nodes=nodes,

          edges=edges_df).

In edges_df, the node indices are specified as 'i' and 'j', and the connectivity weight is in the optional column 'weight'. Additional columns in the data frame can further customize the figure (see the Network Visualizations Are Highly Customizable section below). The default column names 'i', 'j' can be changed with the “edge_columnnames” keyword argument. The keyword argument “edge_weights” specifies the weight of edges.

#### Combining the three components.

The three components are, by definition, interrelated to each other, with nodes being specified in the template space and edges referencing nodes. However, in *NetPlotBrain*, each component can be specified and customized separately (see Figure 1 for a graphic of how the three components can be specified and how they combine). Note that, if using *NetworkX*, it is also possible to input the nodes and edges simultaneously using the “network” keyword argument (see Integration With Other Software section below).

### Network Visualizations Are Highly Customizable

The number of options to customize the figures is extensive and will not be presented in full (see the online documentation). Here, we will discuss four main aspects relating to customization: (a) the naming convention of keyword arguments, (b) the display alternatives for templates and nodes, (c) the high-level interface for specifying node or edge properties, and (d) the viewing options.

#### The naming convention of keyword arguments.

There are a lot of possible keyword arguments that can be used in *NetPlotBrain*. Generally, we try to preserve the option name in the software we use, such as *Matplotlib* (e.g., alpha for transparency). However, since there are multiple components, the keyword arguments follow the following convention: <component>_<option>. This format entails that, for example, arguments such as “template_alpha”, “node_alpha”, and “edge_alpha” will each modify the transparency of each respective component as expected from the *Matplotlib*’s alpha argument. The full list of keyword arguments can be found at netplotbrain.org/api.

#### Display alternatives for templates and nodes.

**Figure 2.**
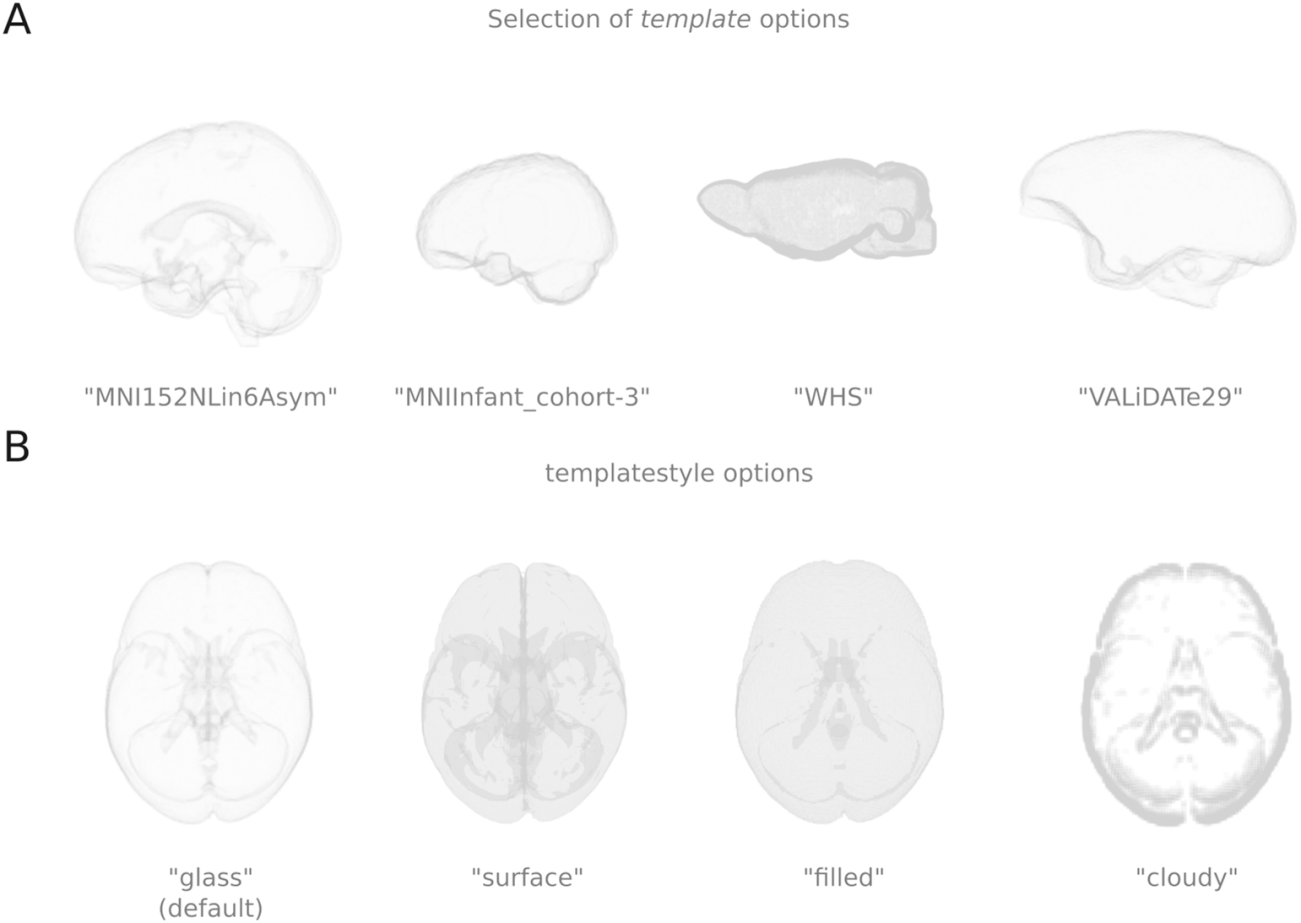
Display options for templates. (A) A selection of options from *TemplateFlow*. (B) Different template styles to render the template.

All templates with NIfTI anatomical files on *TemplateFlow* can be specified in the “template” keyword argument. A change in specification from this single argument allows for a diverse number of templates to be shown, including age ranges and species (see Figure 2A). For templates, there are different methods that generate varying background styles. The keyword argument “template_style” governs the style. Figure 2B illustrates four current template styles: glass, surface, filled, and cloudy.

The different templates generate approximate surfaces or plot the voxels in 3D space. Scikit-image is used in various ways to create the styles for glass, surface, and cloudy by rendering a surface or identifying borders. Additional arguments can be supplied to tweak the surface or border generations for the skimage.segmentation.slic function, which is used to identify the borders in the glass template style. The filled style plots each voxel. This style can increase memory consumption and, consequently, reduce the rendering speed. However, adding the keyword argument “template_voxelsize” and specifying a higher value than the input image will significantly increase performance. In addition, there is also the possibility of specifying hemispheres for the figure. In such instances, only the connections within that hemisphere are shown.

**Figure 3.**
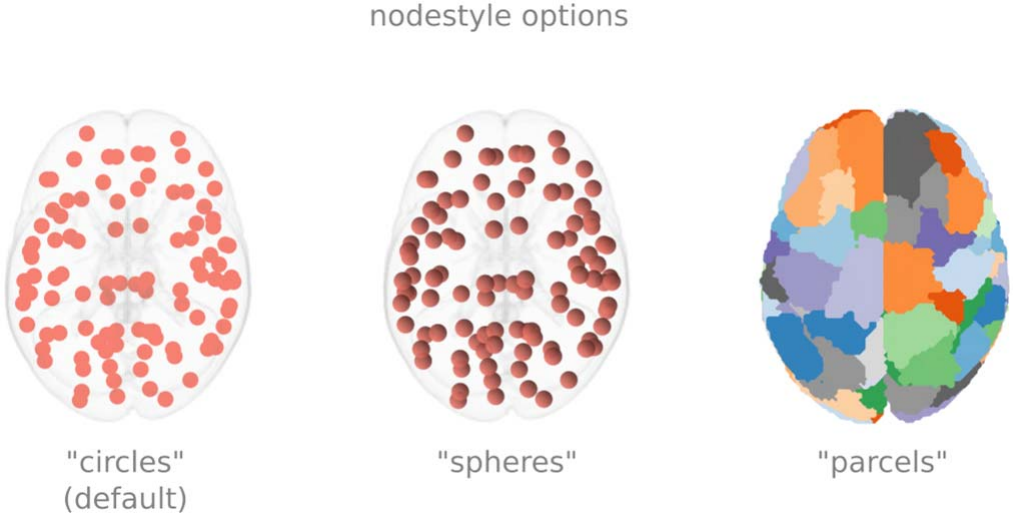
Display options for nodes. The figure shows the same 100 nodes plotted as circles, spheres, and parcels. If the parcels option is chosen, the nodes need to be specified using a NIfTI image.

For nodes, there are currently three options for node styles, which can be specified via the “node_style” keyword argument. The first node style is circles, which plots a circle marker. The second is spheres, which generates a 3D sphere in the image. The third is parcels, which, using scikit-image, generates a surface per parcel (see Figure 3).

For edges, straight lines are drawn between the nodes' centroids. Properties of the edges, such as size and color, can be specified (see the next section).

#### High-level interface.

This section will demonstrate how our high-level interface has been designed to easily specify different properties in the figure. This interface entails that data that specify figure properties can be additional columns in the node or edge input data frames. For example, network measures for both nodes and edges could be included in the data frame and used to set the color, shape, highlighting, and so on. Thus, the input data frame for nodes or edges could be the following:

nodes_df = pd.DataFrame(data={'x':                […],

                'y':                 […],

                'z':                 […],

               **'community':     [1,   1,  2  …],**

               **'participation_coef': [0.5, 0.2, 0.8, …]**}).

edges_df = pd.DataFrame(data={'i':                […],

                'j':                 […],

                'weight':              […],

               **'edge_betweenness': [0.3, 0.1, 0.9,…]**}).

It is then possible to specify properties such as color, size, and highlighting. This feature entails having to specify only the column name for the property. For example, the code to specify the size of nodes by the “participation_coef” and color it by the “community” is the following:

netplotbrain.plot(nodes=nodes_df,

          node_color='community',

          node_size= participation_coef').

**Figure 4.**
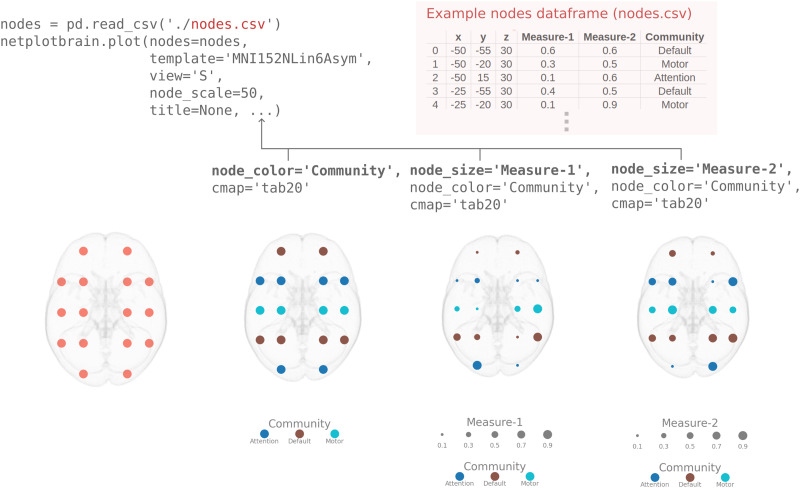
Minimal example showing the high-level interaction with data frame input to customize. The top left area shows code that first loads a .csv file and then calls netplotbrain.plot, passing in information about the nodes. The top right area shows the first few lines of the .csv file, illustrating the mandatory ‘x’, ‘y’, and ‘z’ coordinate columns but also additional measures “Measure-1” and “Measure-2” (e.g., centrality measures) as well as “Community” (the community affiliation). The bottom of the figure shows how the high-level interface works by calling the column names in the loaded .csv file. The code snippets above each figure show what is added to the top left code snippet to change the figure. Figure legends are automatically generated.

See Figure 4 for a minimal example of how to easily color and resize a subset of nodes based on the nodal information stored in the *pandas DataFrame*. See Figure 6 for an example of the highlighting functionality.

Additionally, suppose nodes are specified with an argument that is not a data frame (e.g., a dictionary to specify a *TemplateFlow* atlas). In that case, it is possible to add additional information about the nodes or edges using the keyword arguments “nodes_df” or “edges_df”:

nodes_tf = {'template':     'MNI152NLin2009cAsym',

       'atlas':       'Schaefer2018',

       'desc':        '400Parcels7Networks',

       'resolution':                      1}

nodes_df = pd.DataFrame(data={'community':   [1, 1, 2 …],

               'participation_coef': [0.5, 0.2, 0.8, …]})

netplotbrain.plot(nodes=nodes_tf,

          nodes_df=nodes_df).

#### Viewing options.

*NetPlotBrain*’s intended use is to generate multiple subplots of the same brain for display purposes (e.g., in journals) rather than to provide an interactive interface. Teoretically, the figures can be manually rotated, but this is often sluggish using *Matplotlib*. Thus, *NetPlotBrain* is designed to generate multiple panels of the figure from different viewing angles. There are multiple ways this can be specified:

1. Prespecifying viewing angles and combinations of angles
2. Generating a series of rotating images between two views
3. Specifying the angle and elevation

As for prespecified viewing angles and combinations of angles, these include single letters, combinations of letters for multiple plots, or preset combinations. The available letter alternatives that assign the angles are the following:

'S': superior

'I': inferior

'P': posterior

'A': anterior

'L': left

'R': right

's': spring layout (See the Integration With Other Software section below for more about this option.)

For example, to view an image from the anterior perspective, the letter “A” can be provided:

netplotbrain.plot(template=…,

          nodes=…,

          edges=…,

          **view='A'**).

Alternatively, for the superior preset angle, the letter “S” can be provided:

netplotbrain.plot(template=…,

          nodes=…,

          edges=…,

          **view='S'**).

A string of multiple letters can also be specified to generate multiple subplots. For example, the following will generate three panels (i.e., left, superior, and right preset angles):

netplotbrain.plot(template=…,

          nodes=…,

          edges=…,

          **view='LSR'**).

Finally, generating multiple rows of subplots is possible by specifying a list of preset angles. For example, the following will generate two rows and three columns of figures:

netplotbrain.plot(template=…,

          nodes=…,

          edges=…,

          **view=['LSR', 'AIP']**).

There are also six combinations of different viewing angles that can all be specified using the string “preset-”:

netplotbrain.plot(template=…,

          nodes=…,

          edges=…,

          **view='preset-6'**).

**Figure 5.**
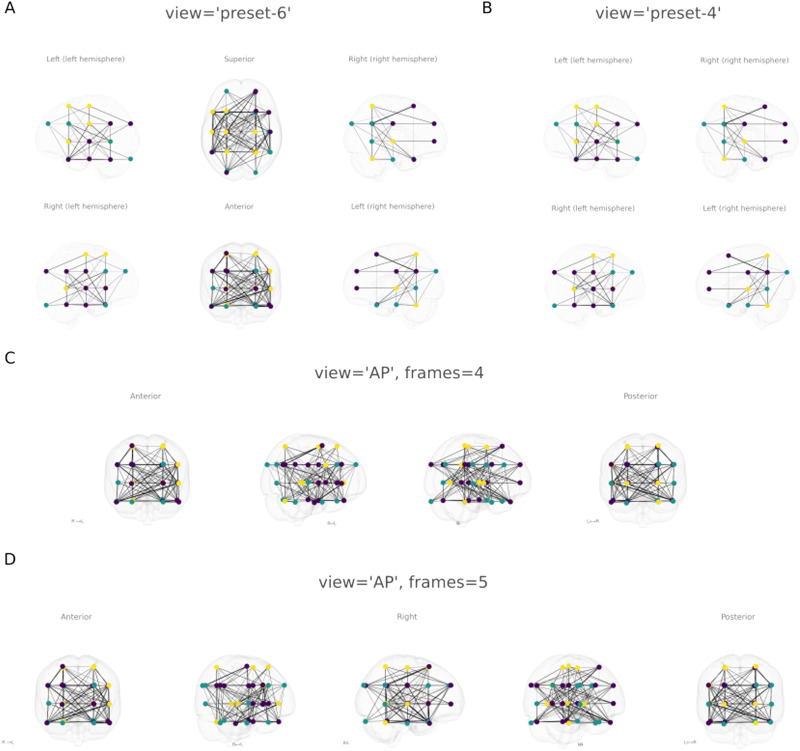
Examples of different views. (A) Multi-panel preset of six different viewing angles (view = 'preset-6'). (B) Same as panel A but for four viewing angles (view = 'preset-4'). (C) Anterior-view to posterior-view rotating for four frames. (D) Same as panel C but for five frames.

See Figure 5A and 5B for examples of the different views. See https://netplotbrain.org/preset for all the preset angle combination alternatives.

The second way to define views is to specify two preset views (e.g., view = 'AP') and thereafter specify a “frames” keyword argument, which will define how many subplots will be generated, rotating between the two specified views:

netplotbrain.plot(template=…,

          nodes=…,

          edges=…,

          **view='AP'**,

          **frames=3**).

Figure 5C and 5D show the difference between specifying four and five rotating frames.

The final viewing version is to specify the rotation (along the XY axis) and the elevation (along the XZ axis) as a 2-tuple. This option is useful only when wanting to capture a default angle outside of the preset angles.

Additionally, while *NetPlotBrain* aims to be simple to use with a single plot function, many visualization options are available. We have documented all the options at https://netplotbrain.org/api and placed them in different categories to assist in finding the correct keyword argument. Finally, we have placed many figures in the netplotbrain.org/gallery to provide the users with examples of the different possibilities.

### Integration With Other Software

In this section, we discuss the *TemplateFlow* integration advantages and detail two additional ways other software can integrate with *NetPlotBrain*.

The *TemplateFlow* integration promotes the usage of consistent spaces in the figure. In previous sections, we have discussed how this integration occurs, but here we will detail the benefits. Ciric et al. (2022) argue that, in many neuroimaging articles, authors have not been precise in specifying which template they have used and have been reporting vague phrases such as “in MNI space” instead. Given the ambiguity in reporting the methods, it is likely and understandable that researchers have inadvertently plotted incorrect nodes or background templates in their network visualizations. This integration means that the nodes may have come from one MNI space and the template from another. There are generally no checks to ensure that the correct background template is in the same space as the nodes. Guidelines for well-designed user interfaces, including preventing the user from making errors (Mazumder & Das, 2014), have been considered in *NetPlotBrain*. Our solution to minimize possible user error is to utilize *TemplateFlow*’s API in the background to find the background templates and atlases. Instead of including a default template, the user must know which template they used and specify it. This implementation encourages the user to provide the correct input without the required expert knowledge to make the correct decision. However, this design choice does not impact the learning curve too much, as output in BIDS derivatives from preprocessing software, such as *fMRIPrep* (Esteban et al., 2019), includes the template space name in the preprocessed data’s file names. Thus, we believe that *NetPlotBrain* promotes accuracy by striking a good balance between usability and required knowledge.

In addition to *TemplateFlow*, *NetPlotBrain* supports two additional software packages: *NetworkX* (Hagberg et al., 2008) and network-based statistics (NBS; Zalesky et al., 2010). *NetworkX* can specify the nodes, edges, and their respective network properties. The integration with *NetworkX* is twofold. First, it is possible to input a *NetworkX* network (a *Graph* object) to *NetPlotBrain* that contains the nodes and edges information. To do this, the “network” keyword argument is used:

**nx_network = nx.Graph(…)**

netplotbrain.plot(template=…,

          **network=nx_network**).

**Figure 6.**
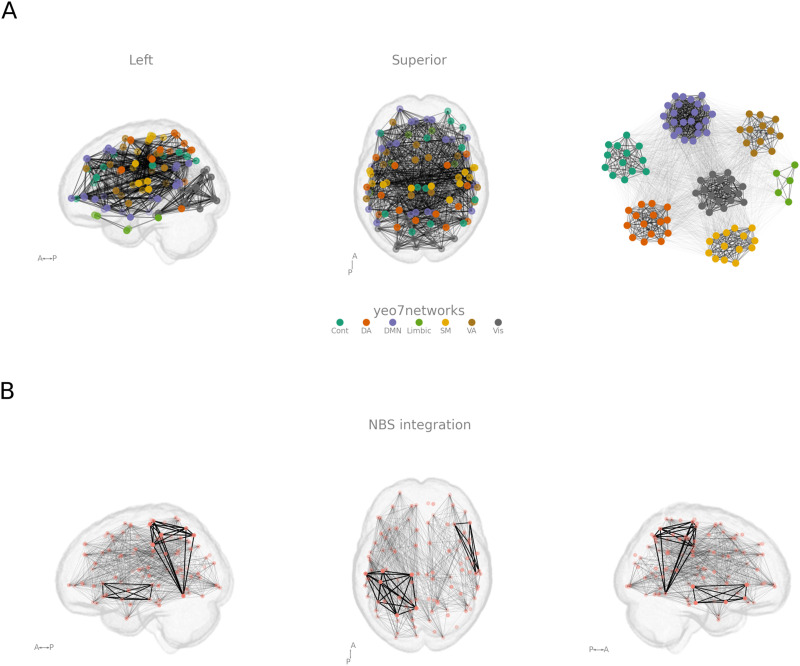
Example of the highlighting functionality. (A) An example of how to easily add a spring layout plot containing all the high-level interface features. In this example, the “view” keyword argument is 'LSs'. (B) An example of the highlighting of clusters of edges that were significant. The code for both examples can be found on netplotbrain.org/gallery.

For this approach to be successful, the *Graph* object needs to have the node attributes x, y, z (or different attributes, if passing a “node_columnnames” keyword argument). Any other attribute in the *nx.Graph* object can be used in the high-level interface (see Figure 6A).

The second integration with *NetworkX* is its implementation of spring layout figures by entering a lowercase “s” as a value passed to the “view” keyword argument. This argument will create an additional subpanel and plot a spring layout figure with all the size, color, and highlight features preserved. This integration allows the figure to include brain and spring layouts with the same color and size specifications (see Figure 6A and netplotbrain.org/gallery/spring_layout for a code example).

Another current integration is with Python implementations of network-based statistics. NBS is a common way to identify the presence of significant clusters (Zalesky et al., 2010). Python implementations of NBS can be found in *pybct*, the Python implementation of the Brain Connectivity Toolbox. With the output from the NBS functions being passed to the “highlight_edges” keyword argument, the significant clusters of the network can be easily visualized (see Figure 6B and netplotbrain.org/gallery/nbs for a code example).

Additional integrations with other software and specifications will be added over time. One example is the connectivity matrix BIDS specification that is currently being drafted. Once finalized, additions to *NetPlotBrain* can include ways to plot group averages or differences by specifying only the relevant derivatives directory.

## DISCUSSION

*NetPlotBrain* is a visualization tool developed in Python 3.9+ to plot networks onto a brain. The main benefits of *NetPlotBrain* are (a) being easy to use while offering a wide array of customization options, by means of its high-level interface; (b) ensuring the accuracy of plots using its integration with *TemplateFlow* (Ciric et al., 2022); and (c) being integrated with other network infrastructures for neuroimaging and Python, such as *NetworkX* and *pyBCT*’s implementation of network-based statistics. One additional advantage of *NetPlotBrain*’s integration with *TemplateFlow* (Ciric et al., 2022) is that when new templates and atlases are added to *TemplateFlow* over time, they become immediately available for visualizing in *NetPlotBrain*.

As discussed in the Introduction, plotting networks can be difficult. With *NetPlotBrain*, a wide number of network-related plots can be generated for a wide variety of use cases. These uses include (but are not exclusive to) displaying a parcellation, showing an entire connectivity matrix on the brain, showing the edges of a single community, highlighting results based on network theory measures, highlighting nodes of interest (e.g., seed regions), showing seed-seed connectivity, displaying significant clusters, plotting differences between groups, conducting exploratory studies, and pairing results both on spatial coordinates and on spring layout figures.

On the documentation page of *NetPlotBrain* (https://netplotbrain.org), there is a gallery of different plots that can currently be created using *NetPlotBrain*. Each example leads to a notebook of how to create these plots. At the top of each notebook, there is a link to open the document in an interactive notebook on mybinder.org to allow users to interact with the examples.

While *NetPlotBrain* assists researchers to easily create high-quality visualizations, clearly communicating the intended message can still require tweaking and consideration. For example, the plots in Figure 5 and Figure 6A are not constructed to convey information about any systematic pattern in the edges but rather to demonstrate different visualization options. Edge information on such figures can be hard to interpret, which is one of the reasons why we have added the option to highlight nodes and edges (e.g., Figure 6B). Creating the clearest desired figures may require considering how to utilize the different visualization arguments in *NetPlotBrain*. In sum, while the figures can be easily created, some care is still needed from the user in order to communicate the intended message to the readers.

### Limitations

*NetPlotBrain* has been conceived as an effective tool for network neuroscience communication, mostly to generate static figures inserted in scientific manuscripts or presentations. Therefore, the design has not considered user-controlled rotation of the figure as a feature. We would recommend visualizing multiple subplots instead of rotating the figure.

Additionally, the current version of *NetPlotBrain* renders only NIfTI files. This current limitation entails that surfaces (e.g., GIFTI or CIFTI file formats) cannot be specified. However, this feature will be implemented in a later version. Since later versions of the software will add support for additional file formats after print, an up-to-date table of the features available in *NetPlotBrain* is available at https://www.netplotbrain.org/features/.

## METHODS

### Installation

*NetPlotBrain* can be installed from PyPi with

pip install netplotbrain

or directly from https://github.com/wiheto/netplotbrain (Thompson et al., 2023) to update to the latest development version. It is recommended to have at least 2 GB RAM to use *NetPlotBrain* effectively.

### NetPlotBrain Dependencies

*NetPlotBrain* is written in Python 3.9.x+ and utilizes *TemplateFlow* (Ciric et al., 2022), *Matplotlib* (Hunter, 2007), *pandas* (McKinney, n.d.), *NumPy* (Harris et al., 2020), *scipy* (Harris et al., 2020), *nibabel* (Brett et al., 2020), *NetworkX* (Hagberg et al., 2008), *scikit-image* (van der Walt et al., 2014), and *PIL* (Clark, 2015).

### Workflow

Using *NetPlotBrain* requires only the netplotbrain.plot function. This function takes in information from up to three sources: the template, the nodes, and the edges. The high-level interface allows each component to be tweaked and customized to display the information needed (e.g., node size, color, or highlighting of significant clusters).

### Data Used

Our examples use the data available on *TemplateFlow* (Ciric et al., 2022), including the Schaefer atlas (Schaefer et al., 2018) and the Yeo 7 community assignments (Yeo et al., 2011). Aside from this, all data included in the examples are either random or pseudorandom. The code used to create data is included in the scripts and the pseudo data are included in the *NetPlotBrain* package.

### Code Examples in the Text

All code examples assume that *NetPlotBrain* has been imported (i.e., import netplotbrain has been run). Whenever “…” is included, that indicates some missing code not directly relevant to the point being made in the example. If a certain part of the code is critical for the example, it is presented in bold. Further, in the main text, keyword arguments are encased in double quotation marks. Input values to keyword arguments are encased in single quotation marks.

## ACKNOWLEDGMENTS

We would like to thank Oscar Esteban for providing valuable comments on an early draft of the manuscript.

## DATA AND SOFTWARE AVAILABILITY

*NetPlotBrain* was developed as an open-source software released under Apache 2 license. The code used to plot the figures shown or mentioned above can be found at https://github.com/silviafan/netplotbrain-figures (for the figures in this article) or/and https://netplotbrain.org/gallery/ (for updated documentation).

## AUTHOR CONTRIBUTIONS

Silvia Fanton: Software; Visualization; Writing – original draft; Writing – review & editing. William Hedley Thompson: Conceptualization; Methodology; Project administration; Software; Supervision; Visualization; Writing – original draft; Writing – review & editing.

## FUNDING INFORMATION

Silvia Fanton, European Union’s Horizon 2020 research and innovation programme under the Marie Skłodowska-Curie Grant Agreement (https://dx.doi.org/10.13039/100018694), Award ID: 764860.

## TECHNICAL TERMS

High-level interface: Interaction occurs through more abstract specification of components. Easier to use compared with low-level interfaces.
Data frame: Data structured into columns and rows, often saved as .csv or .tsv files.
*NetPlotBrain*: A Python package to plot *net*work *plot*s onto *brain*s.
*MatPlotLib*: A standard Python package to plot figures.
*Pandas*: A standard Python package for data frames.
*TemplateFlow*: Online repository for brain templates that includes humans, infants, rodents, and atlases.
Python dictionary: Data structured with key and value pairs.
Spring layout: A type of network visualization.

## Supporting Information

https://github.com/wiheto/netplotbrain
